# Adsorption and bacterial performance of Nd_2_O_3_ modified Ag nanoparticles with enhanced degradation of methylene blue

**DOI:** 10.1038/s41598-024-57226-4

**Published:** 2024-04-30

**Authors:** Mohamed Tharwat Elabbasy, M. A. El-Morsy, Nasser S. Awwad, Hala A. Ibrahium, A. A. Menazea

**Affiliations:** 1 Pathology Department, College of Medicine, Ha’il University, 55476 Ha’il, Saudi Arabia; 2 Physics Department, Plasma Technology and Material Science Unit, College of Science and Humanities in Al-Kharj, Prince Sattam Bin Abdulaziz University, 11942 Al-Kharj, Saudi Arabia; 3 Physics Department, Faculty of Science, University of Damietta, New Damietta, 34517 Egypt; 4 Department of Chemistry, College of Science, King Khalid University, P.O. Box 9004, 61413 Abha, Saudi Arabia; 5 Department of Biology, College of Science, King Khalid University, P.O. Box 9004, 61413 Abha, Saudi Arabia; 6 Spectroscopy Department, Physics Research Institute, National Research Centre, Dokki, 12622 Giza Egypt

**Keywords:** Optical, Silver nanoparticles, Neodymium oxide, Water treatment, Chemistry, Materials science, Physics

## Abstract

Our study focused on the optical behavior, methylene blue (MB) dye degradation potential, antibacterial performance, and silver and trioxide mineral interaction with different bacterial species. We found that the addition of silver nanoparticles (Ag NPs) to neodymium oxide (Nd_2_O_3_) resulted in a significant response, with an enlargement of the inhibition zone for bacterial species such as *Staphylococcus aureus* and *Escherichia coli.* Specifically, the inhibition zone for *S. aureus* increased from 9.3 ± 0.5 mm for pure Nd_2_O_3_ to 16.7 ± 0.4 mm for the Ag/Nd_2_O_3_ nano-composite, while for *E. coli*, it increased from 8.8 ± 0.4 mm for Nd_2_O_3_ to 15.9 ± 0.3 mm for Ag/Nd_2_O_3_. Furthermore, the optical behavior of the composites showed a clear band-gap narrowing with the addition of Ag NPs, resulting in enhanced electronic localization. The direct and indirect transitions reduced from 6.7 to 6.1 eV and from 5.2 to 2.9 eV, respectively. Overall, these results suggest that the Ag/Nd_2_O_3_ nano-composite has potential applications in sensor industries and water treatment, thanks to its enhanced optical behavior, antibacterial performance, and efficient MB degradation capabilities. In terms of MB degradation, the Ag/Nd_2_O_3_ mixed system exhibited more efficient degradation compared to pure Nd_2_O_3_. After 150 min, the MB concentration in the mixed system decreased to almost half of its starting point, while pure Nd_2_O_3_ only reached 33%.

## Introduction

In the modern domain, the routine usages of cosmetics, dyes, and chemicals are amplified. Thus, global water resources were contaminated via leftover chemicals and dyes. This contamination directly affected all living creatures^1–6^. The existence of dye in water is toxic, and also prevent sunlight passing through water which influences aquatic organisms. Henceforth, appropriate dye removal is essential before its release to water bodies. decomposition of dye pollutants into non-toxic ingredients is a challenge^7^. Advanced Oxidation Processes (AOPs) for removing pollutants embrace several methods, for instance, ozonation, photocatalysis, and electrochemical oxidation. In this study Methylene blue (MB) dye was decomposed upon Ag/Nd_2_O_3_ utilization. The MB dye contamination is spread widely owing to its usage in dyeing and printing textile industries^8^. The azo functional group (–N=N–) which exists in MB structure is considered carcinogenic^9^. Additionally, methylene blue (MB) inhalation affects digestive system pains, respiratory system sicknesses, as well nervous systems disorders^10^. Saravanan et al.^11^ develop a dye removal Photocatalytic process by replacing UV radiation instead with visible electromagnetic waves. Photocatalytic degradation is influenced by band-gap, surface area, and grain size, and amount of material^12^. Metallic nano-scale metals possess special physical and chemical activity that push in exploiting them in numerous fields such as electronics, textile, medical, etc. catalytic potential of metals recommends its usage in the degradation of toxic dyes^7^. Further, metallic insertions belong to exceptional electric, magnetic and optical performances. Semiconductor catalysts are preferred in the process of photocatalytic degradation of wastewater due to several advantages. Firstly, they are cost-effective. Secondly, they are non-toxic. Thirdly, they possess adaptable characterization that could be altered through doping, size adjustment, or sensitizers. Fourthly, they enable a multi-electron relocation process. Lastly, they can be utilized extensively without significant reduction in their photocatalytic efficiency^13^.

The optical and magnetic characteristics of Neodymium oxide nanoparticles have much attention for numerous usages. Indeed, Nd_2_O_3_ is extensively utilized in photonic, luminescent, and thermo luminescent usages^9^. Moreover, grayish blue hexagonal crystal lattice of neodymium oxide (Nd_2_O_3_) offers significant catalytic, electric, coloring, besides additive characteristics^14,15^. Nd_2_O_3_ enhances photocatalytic activity by facilitating the effective separation of photo-generated electrons^16^. Casillas et al.^17^, studied the photo-catalytic degradation of diclofenac in aqueous system catalyzed with Al_2_O_3_–Nd_2_O_3_ oxides and the effectiveness linked to the Nd_2_O_3_ contribution. Studying silver doped neodymium oxide nano-composite is favored owing to its inertness, non-toxicity, high surface area^10^, which are different from their macro-scaled equivalents. Additionally, the energy gap could be narrowed by modifying surface defects by forming nano-composites^17^. A research revealed that CuO nanoparticles doped with Nd_2_O_3_ had a remarkable photocatalytic efficacy, reaching up to 90.8% during a duration of 80 min, accompanied with a degradation rate of 0.0227 min^−1^^18^. On the other hand, from 90 to 100% degradation was detected for several dyes by silver NPs. Also, the advantage of NPs usage in dye degradation process is time saving with lacking any hazardous chemical^7^. Such usages strongly depend on the morphological features, and crystal structure of silver NPs^19^. The incorporation of AgNPs into Nd_2_O_3_ improves the efficiency of photocatalysis due to many factors. To begin with, doping involves the incorporation of distinct metallic elements into the structure of the photocatalyst, resulting in a fundamental modification of its physical and chemical characteristics. This allows for the inclusion of a larger quantity of photogenerated electrons and holes, so effectively adjusting the reaction towards visible wavelengths^20^. Furthermore, AgNPs enhance the photocatalytic activity driven by ultraviolet light by facilitating the separation of electrons and holes. Additionally, AgNPs also stimulate the photocatalytic activity driven by visible light via the localized surface plasma resonance (LSPR) of AgNPs^20^. Hence, the addition of AgNPs to Nd_2_O_3_ doping might augment the photocatalytic efficiency by broadening the spectrum of light absorption and facilitating the segregation of photo-generated charge carriers^20,21^. Further, the plasmon excitation boosts optical field response^19^. Lately scientists notify AgNPs as a recommended degradation agent of methylene blue^7^. Owing to the previous survey; Nd_2_O_3_, and Ag NPs are recommended for effective dye removal strategy. The microstructure, optical, and antibacterial behavior is examined.

Bacteria, fungus, and plants are effectively used in biological methods to degrade different textile dyes. In order to harness the capabilities of microbial consortia for improving dye degradation, it is important to consider the wide range of enzyme activation that might occur within a culture. The drawbacks of this approach include the need for prolonged retention times for the conversion of a functional molecule and its complete mineralization. The combination of photocatalysis for dye removal and antibacterial activity for water treatment is more effective and will reduce the cost of the water treatment process^22^.

The objective of this paper is to illustrate the influence of Photocatalytic properties on the dyes removal capability and assess the adsorption aptitude of Nd_2_O_3_ and AgNPs compositions in environmental applications, particularly water decontamination and antibacterial activity. Additionally, the research aims to examine the optical behavior of the pure Nd_2_O_3_ composition.

## Experimental techniques

### Preparation of Ag/Nd_2_O_3_ NPs

In the first step, 100.0 mL of 0.1 M silver nitrate (99.9%) was mixed by 100.0 mL of 0.1 M neodymium chloride (99.8%) solution. In the second step, 200 mL of 0.1 M thio-urea solution has been added while manipulating the temperature at 80 °C for 3 h on a magnetic stirrer. Throughout the heating process, 1 M NaOH (99%) has been gradually added to obtain the desired alkaline precipitate. After 3 h, the mixture was cooled down to room temperature and then washed with distilled water and ethanol to dissolve any contaminations. Finally, the precipitate was dried at 70 °C for three days. During the stirring and heating process, the following reactions were predicted: Sodium hydroxide was dissolved, giving aqueous sodium cations and hydroxyl anions, as well as silver nitrate and neodymium chloride^13^. The following equations were predicted.

$${\text{OH}}^{ - }_{{({\text{aq}})}} + {\text{ Nd}}^{3 + }_{{({\text{aq}})}} + {\text{ Ag}}^{ + }_{{({\text{aq}})}} \to {\text{AgOH}}_{{({\text{aq}})}} + {\text{ Nd}}\left( {{\text{OH}}} \right)_{{3({\text{aq}})}}$$

$${\text{AgOH}}_{{({\text{aq}})}} + {\text{ Nd}}\left( {{\text{OH}}} \right)_{{3({\text{aq}})}} \to {\text{Ag}}/{\text{Nd}}_{2} {\text{O}}_{{3({\text{s}})}} \downarrow \, + \, 2{\text{H}}_{2} {\text{O}}$$

The resultant product is not water soluble as Nd_2_O_3_ has a very low solubility in water^23,24^.

All raw chemicals have been obtained from Sigma Aldrich. The powder of Ag/Nd_2_O_3_ NPs was exposed to UV–Vis spectroscopy analysis (Lamda-950, PerkinElmer, Germany). The X-ray diffraction (XRD) pattern was attained by an X-ray diffractometer (XRD, X’Pert Explorer, PANalytical diffractometer) equipped with Cu Kα1 radiation (λ = 1.5406 nm) utilizing a originator voltage of 40 kV and current of 35 mA. FTIR analysis have been executed with a spectrometer (iS50 ATR Spectrum-100 FTIR) gotten from PerkinElmer, Germany. High resolution transmission electron microscope (HRTEM, JEM-2100F) has been utilized for the synthesized samples. Additionally, MB degradation evaluation was executed by a spectrophotometer^24^.

The concentration of methylene blue (MB) solution was modified to 20 ppm and the pH was 6. The lighting system used a closed box to emit visible light within the spectrum of 500 watts. Following irradiation, a volume of 3 mL was extracted from the (MB) solution at certain time intervals for analysis using a spectrophotometer. The deterioration potential is determined using the mathematical formula^25,26^. Following the degradation of MB, the resulting precipitated powder was subjected to centrifugation for the purpose of recycling^27,28^.

Preparation of Bacterial Culture: A bacterial culture is created by cultivating bacteria in a media that is abundant in nutrients, hence facilitating their development and multiplication. The culture is placed in a controlled environment to facilitate bacterial growth. Following the incubation period, the culture is examined. If the antibacterial treatment is effective, there should be a region where bacterial proliferation is impeded. The antibacterial activity may be quantified by measuring and using this zone. The antibacterial activity has been tested against both Gram-negative (*Escherichia coli* = *E. coli*) and Gram-positive (*Staphylococcus aureus* = *S. aureus*). The experiment begun with 5 mg/ml starting concentration for the synthesized samples to be examined. The inhibition zone has been measured after 24 h of incubation at 37 °C under visible light.

## Discussion

### XRD

**Figure 1:**
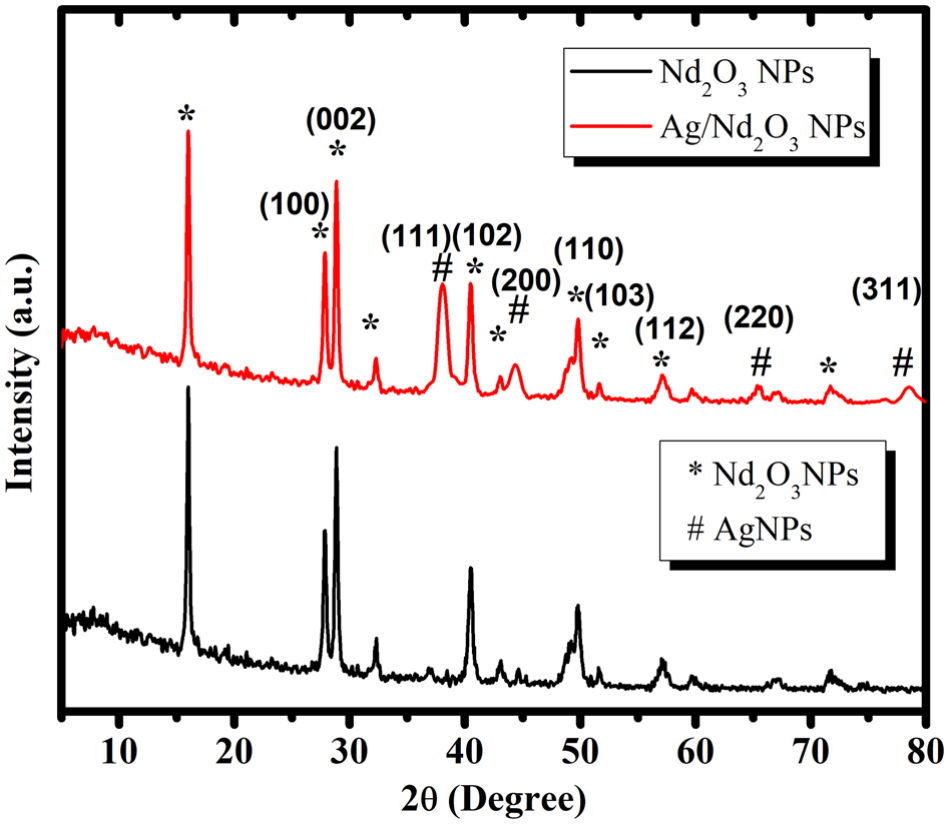
XRD of Pure Nd_2_O_3_ NPs_,_ and Ag/Nd_2_O_3_NPs.

Crystallinity was clarified by XRD analysis that 2θ were introduced in 10–70 scope. For silver, the bands were confirmed its existing, utilizing JCPDS no. 04-0783. Various peaks for silver attained at 2*θ* = 38.3°, 43.9°, 65.1°, and 77.3°, which corresponds to the Bragg’s reflections of the (111), (200), (220), and (311) crystal planes of the face-centered cubic (fcc) structure of metallic silver^29^. Figure 1 also marks peaks for Nd_2_O_3_ according to JCPDS no. 21-0579^30^. The peaks of Nd_2_O_3_ which are at 2*θ* = 27.8, 29°, 40.2°, 49°, 51.6, and 57° ,can be indexed to the (100), (002), (102), (110), (103), and (112), respectively^31^. The resultant patterns do not refer to any major disorders owing to the silver nanoparticle's contribution^17^.

### FTIR

**Figure 2:**
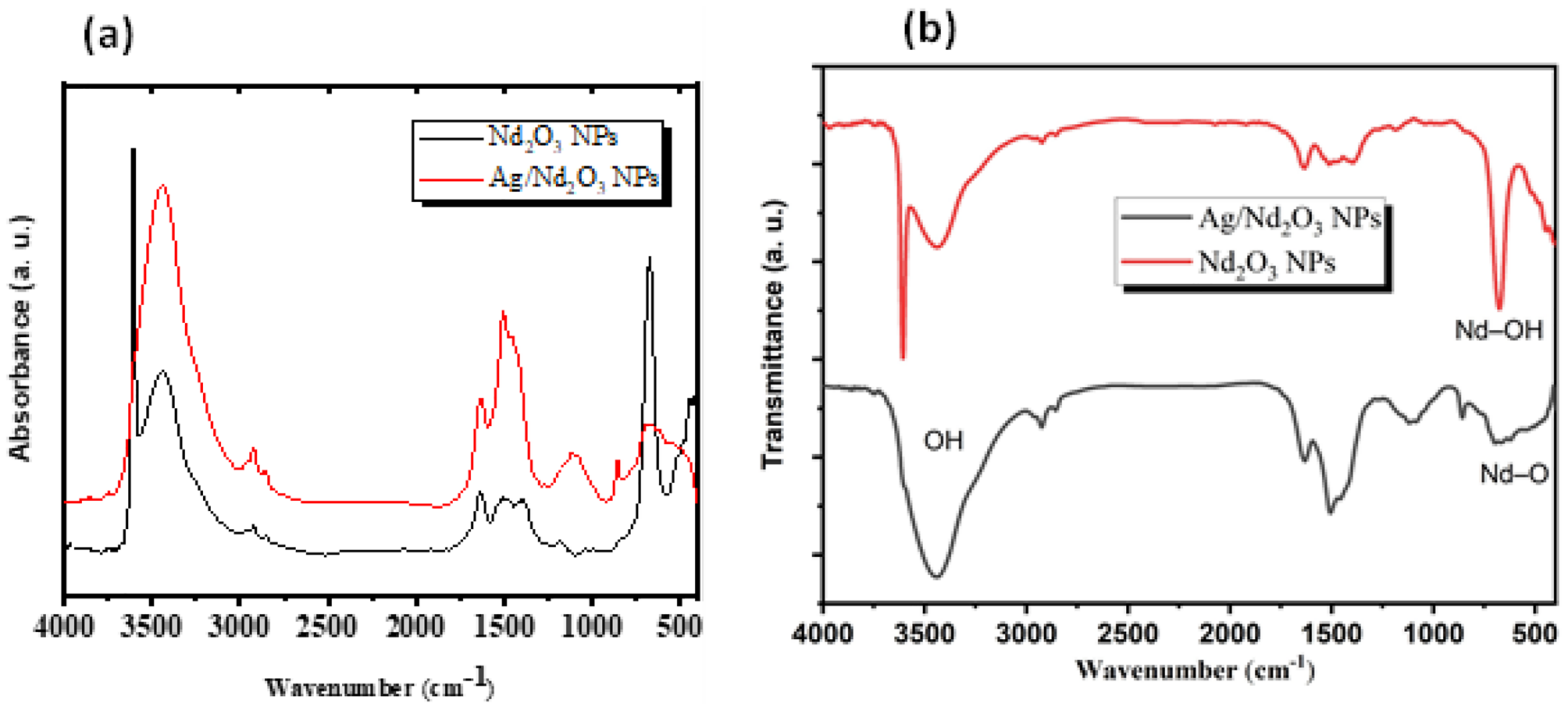
FTIR of Pure Nd_2_O_3_ NPs_,_ and Ag/Nd_2_O_3_NPs.

The FTIR spectra are revealed in Fig. 2. It can be observed that the broadband at 3455 cm^−1^ for pure oxide while adding silver NPs does not cause a noticeable shift that O–H stretching vibration of the –OH group appears at 3448 cm^−1^^32^. The revealing absorption peak at 3554 cm^−1^ is assigned to thio-urea residual N–H stretching^33^. The Neodymium oxide (Nd_2_O_3_) NPs strong band centered at 669 cm^−1^ is attributed to Nd–OH which shows significant diminishing with adding Silver nano-particles^32^. The centered bands at 588 cm^−1^ was because of –O stretching of Nd^30^. The previously mentioned bands confirm Nd_2_O_3,_ Ag/Nd_2_O_3_ chemical composition.

### TEM

**Figure 3:**
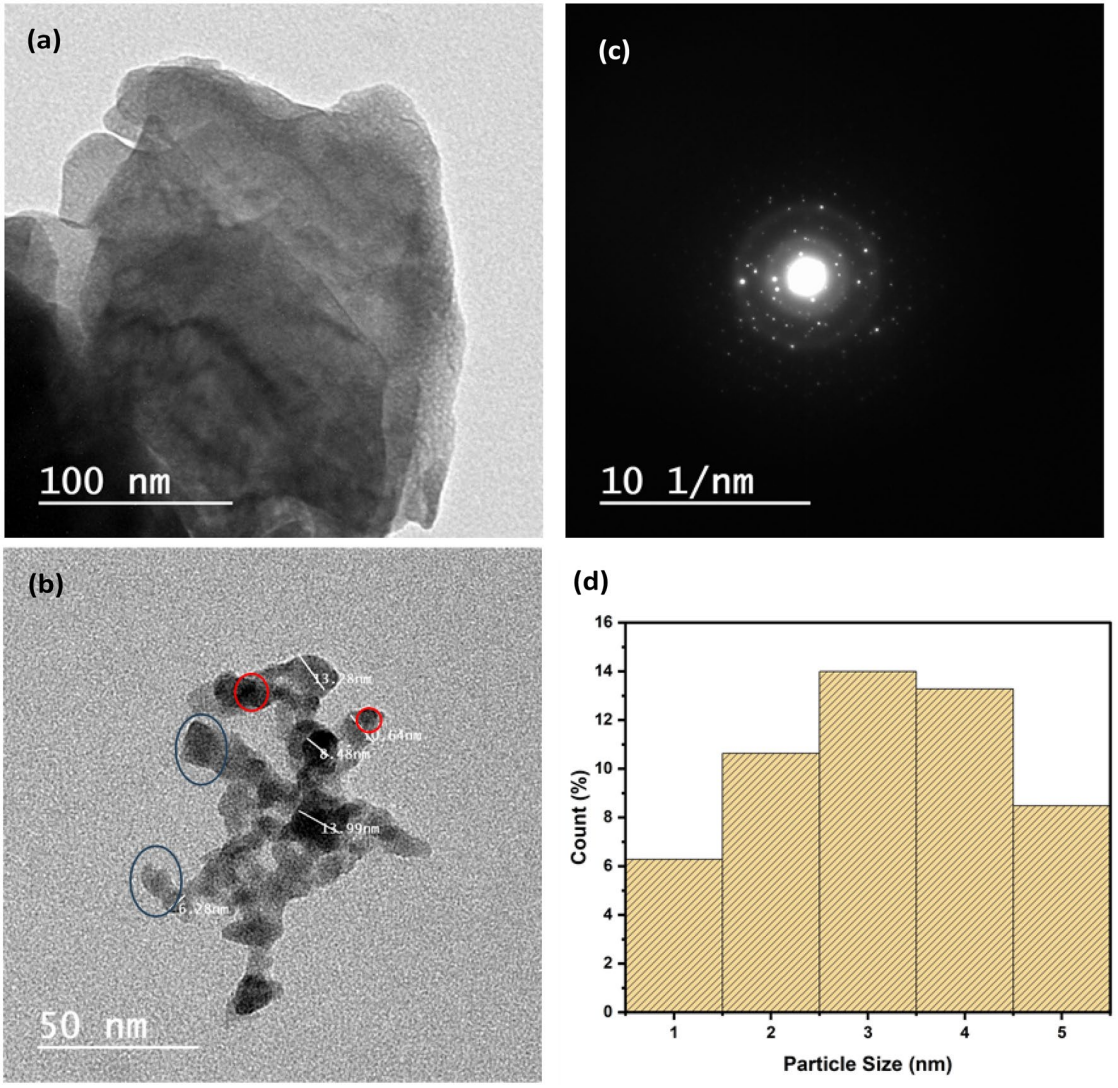
TEM micrographs of (**a**) Pure Nd_2_O_3_ NPs, (**b**) Ag/Nd_2_O_3_NPs, (**c**) saed pattern for Ag/Nd_2_O_3_NPs, (**d**) particle size histogram of Ag/Nd_2_O_3_NPs.

Nd_2_O_3_ NPs microstructure is demonstrated in Fig. 3a. Distinct grains for pure Neodymium oxide are appeared, while Silver nanoparticles appeared with well-defined grains upon the Nd_2_O_3_ matrix. Silver nano-particles average grain size is 9.2 nm. As well, Nd_2_O_3_ introduces significant crystalline portions with distinct grain borders. In addition, the circle inserted in the same figure displays fringes of diffraction points; thus, the presence of well-crystallized Neodymium oxide is approved^17^. The displayed Fig. 3b micrograph confirms the good distribution of Ag NPs upon Nd_2_O_3,_ besides the well matching with XRD results_._ The red circles represent AgNPs which is inside the surface of Nd_2_O_3_ which is represented in blue circles. Figure 3c represent saed pattern for Ag/Nd_2_O_3_NPs and Fig. 3d obtained the particle size histogram of Ag/Nd_2_O_3_NPs.

### Optical study

Studying the optical characteristics of nano-composite have an excessive curiosity in order to discover their proper usage. The optical characteristics of Nd_2_O_3_ and Ag/Nd_2_O_3_ nanoparticles were inspected by UV–Vis spectroscopy in wavelengths ranging from 200 to 1100 nm. The band at 244 nm is for Nd_2_O_3_ NPs that allied an interchange of electrons among the valence and conduction orbits^32^. The optical band gap energy has been assessed by mode suggested by Wood and Tauc. Aiming for optically induced transition understanding, optical band-gaps are obtained from UV absorption spectra. The technique is based on the absorption of a relatively high energetic photon in comparison to bandgap energy. Briefly, there are two types of optical transitions based on absorption edge, they are direct and indirect transitions. Both transition types involve an interaction of the valence band with an electromagnetic wave.

**Figure 4:**
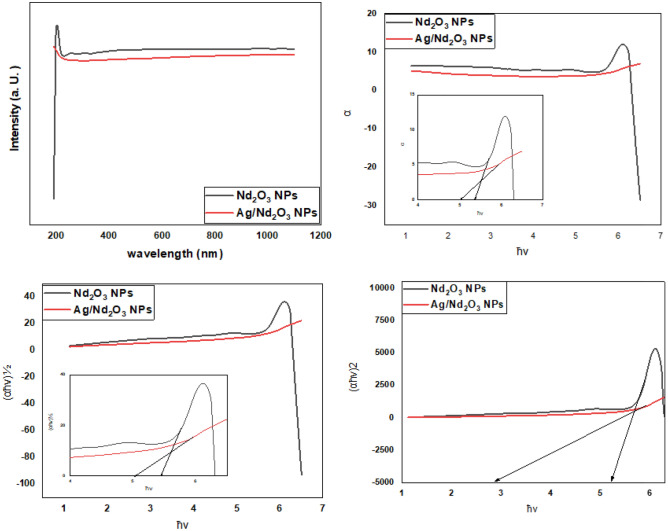
Optical behavior of Pure Nd_2_O_3_ NPs, and Ag/Nd_2_O_3_ NPs.

The Optical absorption coefficient (α) is calculated by Beer–Lambert’s equation^34^. After that, the absorption coefficients (α) plot against (hν) in Fig. 4, showing absorption edge shift was obtained due to silver assimilation. The Eg values were almost 6.8 e.V for pure Neodymium oxide (Nd_2_O_3_) NPs^32^. On the other hand, Ag NPs insertion causes shifting of λ to lower frequency^35^, besides lowering absorption edge to 6.1 e.V^30^. this confirms the well spreading of silver NPs^36^. As well, the band-gap is estimated from $\alpha hv ={A(hv -Eg)}^{m}$ equation^37,38^, where (hν) is for photon energy, (Eg) is for band-gap, (A) is for band tailing parameter, and (m) direct transition is at m = 0.5, and indirect transition is at m = 2).

Bandgap widened with size shrinkage owing to electron confinement at nano-scale which is famed for the “quantum size effect”. The diminishing of bandgap refers to the perfection in crystallographic ordering by adding Ag NPs that enhance boosting in electronic localization upon nano-composite ingredients^39,40^. Consequently, these interpretations boost these compositions’ usage in sensors industries.

Finally, The refractive index (n) is computed using Dimitrov and Sakka formula as a function of indirect energy band-gap.

1 $$\frac{{n^{2} - 1}}{{n^{2} + 1}} = 1 - \sqrt {\frac{{Eg_{i} }}{20}}$$

In likewise, addition, the refractive index alteration as listed in Table 1 refers to varying packing density^36^. This specifies Nd_2_O_3_ NPs and Ag/Nd_2_O_3_ NPs to be utilized in optoelectronic devices.

**Table 1:** Optical properties of Nd_2_O_3_ NPs and Ag/Nd_2_O_3_ NPs nano-composite.

Composition	Absorption edge (eV)	Band-gap (eV)		n
		Direct	Indirect	
Nd_2_O_3_ NPs	6.8	6.7	5.2	1.56
Ag/Nd_2_O_3_ NPs	6.1	6.1	2.9	1.62

### Methylene blue (MB) degradation assessment by Uv–Vis Absorption Analysis

**Figure 5:**
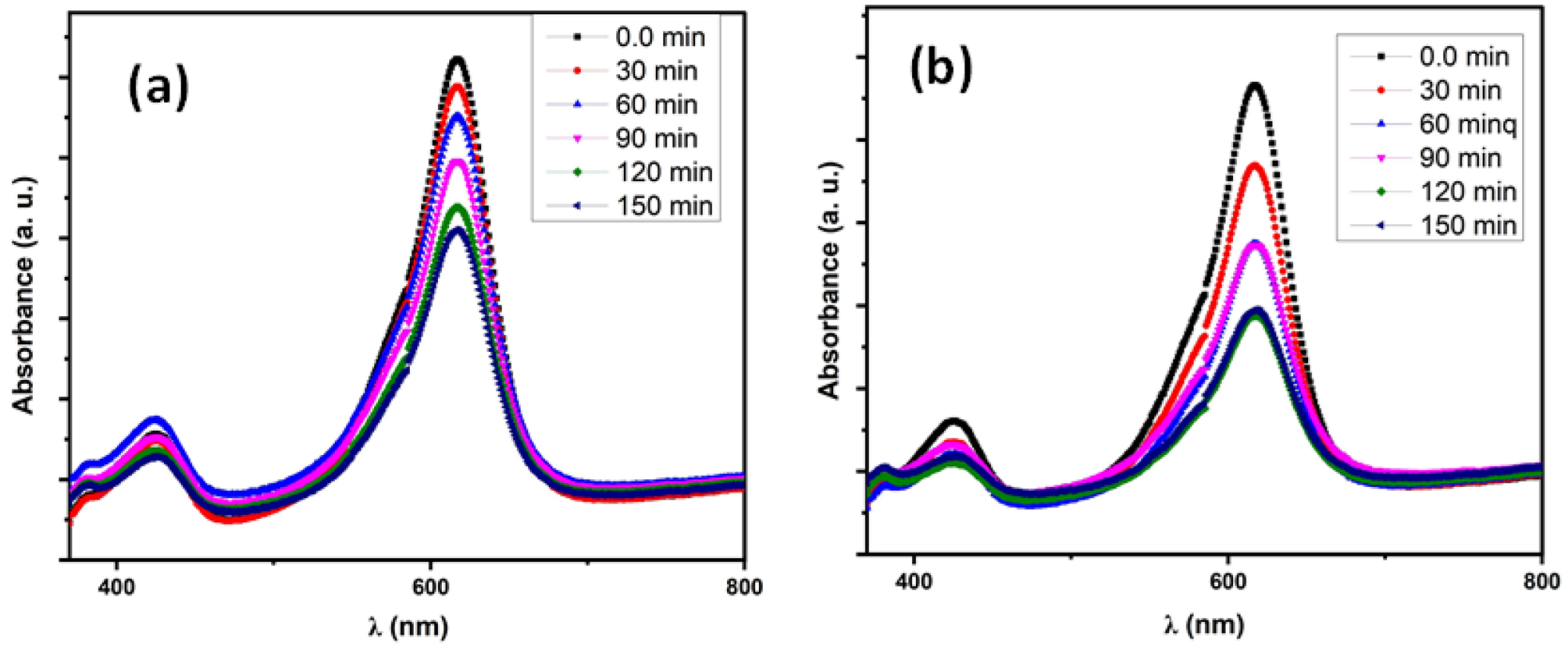
The degradation of MB using (**a**) pristine Nd_2_O_3_ NPs, (**b**) Ag/Nd_2_O_3_NPs.

The characteristic absorbance peak of methylene blue dye was centered at 618 nm. Throughout the irradiation step, the crest in both cases (Nd_2_O_3_ and Ag/Nd_2_O_3_) shows a regular decline in absorbance owing to hypochromic influence. The absorbance of MB solution shows a dwindling pattern with time. this could explain the adsorbing capability of both tested compositions, However, the silver-containing composition exhibits a higher potential for degradation^41^. This may be explained by Nd_2_O_3_ capability to absorb photons that enhance electron excitation, generating positive holes. The electron–hole pairs travel individually to the surface of oxide and lead to redox series with adsorbed species, generating highly reactive oxygen species (ROS). These oxy-species interact with adsorbed contaminations leading to the decomposition process^42^. However, after short time electrons and holes recombine causing a decline in its photo-degradation potential. Thus, decreasing their recombination is executed by metals that act as cationic dopants^42^. Further, in this study degradation assessment of pure Nd_2_O_3_ and AgNd_2_O_3_ nanoparticles Mixed System is executed under visible light. Spectra display a gradual dropping of MB content with irradiation time. The MB degradation efficiency is detected via  peak intensity, thus the dye concentration shows a declining pattern with the time that confirms its efficiency in removing the MB dye. Mixed System shows more efficient degradation owing to silver nano-particle insertion that the MB concentration dropped to half of its starting point at 150 min. Also, The photo-conversion capability using pure Nd_2_O_3_ as a reference photo-catalyst was lower than those of nano-composite, reaching 33% after 150 min of light irradiation, as obtained in Fig. 5^17^. In conclusion, crystallinity, and ROS generation noticeably persuade the surface area, number of active sites, and decline in the particle size that enhances dye decomposition.

The process of degrading MB dye by photocatalysis using Ag/Nd_2_O_3_ nanocomposite consists of many sequential stages: The photocatalytic process starts upon light absorption by the Ag/Nd_2_O_3_ nanocomposite, resulting in the generation of electron–hole pairs. Subsequently, the electrons that are in an excited state undergo a reaction with molecular oxygen, resulting in the formation of superoxide radicals. Simultaneously, the positively charged holes have the ability to directly oxidize the dye or water, leading to the generation of hydroxyl radicals. Subsequently, these radicals exhibit a high level of reactivity, enabling them to break down the dye molecules. Subsequently, the reactive species engage in interactions with the dye molecules, resulting in their fragmentation into smaller, less detrimental constituents^43–45^. This is the first stage at which the perceptible hue of the dye begins to diminish. Ultimately, the ideal outcome is for the dye molecules to undergo full mineralization, resulting in the formation of innocuous chemicals such as water and carbon dioxide^43–45^. The efficacy of this procedure relies on several aspects, such as the characteristics of the nanocomposite, the intensity and wavelength of the light, and the concentration of the dye1. The Ag/Nd_2_O_3_ nanocomposite exhibits enhanced efficacy attributed to the inclusion of silver, which facilitates light absorption and facilitates the dissociation of electron–hole pairs^46,47^.

While previous research mentioned in Table 2 demonstrated significant photocatalytic activity in the removal of MB dye, the composites that were prepared did not exhibit any antibacterial properties. Additionally, the pH of the reaction was not specified in the previous studies. Furthermore, the concentration of dye used was very low. In contrast, our work investigates the photocatalytic activity using a 20 ppm dye concentration and at a pH of 6.

**Table 2:** comparison from different studies about the removal efficiency of different dyes.

Catalyst	Dye degradation	ppm	Time (min)	Refs.
NiCuMoO/rGO	99.2%, MB	10 ppm	80	^48^
LaNdZr_2_O_7_/SnSe	97%, Congo red	–	120	^49^
Zn_0.9_Ho_0.05_M_0.05_O	99.7% MB and 84% MO	5 ppm	60	^50^
NiYHoO	99% MR	5 ppm	60	^51^
NiO–CdO–ZnO	99% for RhB and 98% for MB	5 ppm	60 and 90	^52^
ZnO–CoTe	98.5% MR and 99.8% MB	–	60	^53^

### Anti-bacterial activity

**Figure 6:**
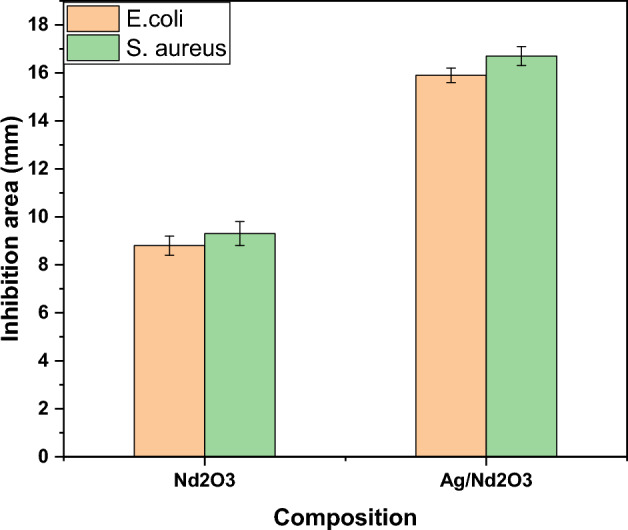
Antibacterial behavior of Pure Nd_2_O_3_ NPs, and Ag/Nd_2_O_3_NPs.

As *E. coli*, and *S. aureus* are the two greatest widespread bacterial species, especially after implant surgery. The introduced performance shows almost doubling an antibacterial efficiency with merging silver into pure Neodymium oxide (Nd_2_O_3_) nanoparticles. For illustration, as shown in Fig. 6, *S. aureus* demonstrates an inhibition zone of 9.3 ± 0.5 mm for pure Neodymium oxide (Nd_2_O_3_) nanoparticles (NPs), 16.7 ± 0.4 mm for Ag/Nd_2_O_3_ nano-composite. *E. coli* inhibition zones reveal bacterial inhibition capacity under the tested inhibitor, it shows 8.8 ± 0.4 mm for Neodymium oxide and 15.9 ± 0.3 mm for Ag/Nd_2_O_3_ nano-composite. The introduced antibacterial pattern is explained by the different nature of gram-positive and gram-negative bacteria, for example, *E. coli* and *S. aureus* have a divergent structure with thicker cell wall peptidoglycan layer in gram-positive than that of the gram-negative species^54^. Derakhshi et al*.*^55^ point to the active antibacterial role of silver nanoparticles in gram-negative bacteria with 800 μg/mL. Additionally, antibacterial composite amount and nature affect the subsequent results. The introduced germicidal valuation is performed in dark, neglecting the plasmonic effect of Ag–NPs. Likewise, The mechanism of antimicrobial activity upon using silver NPs has involved the interaction of silver ions with thiol groups in pathogenic enzymes and proteins, resulting in cell death^56^. Additionally, the effectiveness of released reactive oxy-Species is affected by the inserted silver quantity, size, and distribution^57^. Further, the released oxonium ions (H_3_O^+^) from Nd_2_O_3_ mineral trioxide interrupt the cellular pH, thus bacterial reproduction is disturbed, also does not allow drug-resistant phenomenon occurrence^58^. As well, Wang et al.^59^ studied mineral trioxide/silver nanoparticles nano-composite, finding significant performance of La_2_O_3_ or Ag–La_2_O_3_ nanoparticles in inhibiting *E. coli* and *S. aureus* growth. Therefore, nanomaterials specially mixed systems are highly recommended for biological applications. Nd_2_O_3_ nanoparticles have many mechanisms by which they might impact microorganisms. To begin with, they may cause modifications in the bacterial community's composition by changing the prevalence of uncommon and delicate taxa, therefore reducing the overall effectiveness of the community. In addition, Nd_2_O_3_ nanoparticles have the ability to connect with the cell wall of bacteria, especially in Gram-positive bacteria that have a thick peptidoglycan layer^60–63^. This interaction has an impact on the integrity and function of the cell wall. Nano-Nd_2_O_3_ may disrupt enzyme activity, specifically affecting enzymes that are essential for soil carbon and nitrogen cycle^60^. These enzymes are critical for bacterial survival and function. In addition, like other metal oxide nanoparticles, Nd_2_O_3_ nanoparticles has the capability to produce reactive oxygen species (ROS), which may induce oxidative stress in bacterial cells. Oxidative stress may lead to the impairment of vital cellular molecules and ultimately cause cellular demise^64^. Moreover, upon contact with bacterial cells, the nanocomposite liberates silver ions (Ag_+_), which interfere with the integrity of the cell membrane, eventually leading to the demise of the cells^61,65–67^. The Nd_2_O_3_ nanocomposite exhibits antibacterial characteristics due to the synergistic effects of these coupled processes.

## Conclusion

Structural, and compositional study of pure Neodymium oxide (Nd_2_O_3_ NPs), and Silver (Ag)/Neodymium oxide (Nd_2_O_3_) nano-composite, using XRD, FTIR, and TEM techniques. This study emphasizes optical behavior, MB dye degradation potential of pure Nd_2_O_3_ NPs, and (Ag/Nd_2_O_3_) nano-composite, alongside revealing their antibacterial performance of silver and trioxide mineral interaction with bacterial species. For instance, *S. aureus*, and *E. coli* enlargement of inhibition zone under Ag NPs insertion. *S. aureus* inhibition zone broadened from 9.3 ± 0.5 mm for pure Nd_2_O_3_ to 16.7 ± 0.4 mm for Ag/Nd_2_O_3_ composition, while *E. coli* inhibition zones extended from 8.8 ± 0.4 mm for Neodymium oxide to15.9 ± 0.3 mm for Ag/Nd_2_O_3_. Also, optical behavior displays band-gap contraction with assimilation with Ag NPs, which improves electronic localization. That direct and indirect transitions dropped from (6.7 to 6.1) and (5.2 to 2.9 e.V), respectively. Concerning the methylene blue degradation, Ag/Nd_2_O_3_ shows more efficient Methylene blue degradation than pure Nd_2_O_3_ in that the MB concentration dropped to almost 50% of its starting point after 150 min, while pure Nd_2_O_3_ reached 33% after 150 min of light irradiation. On the other hand, TEM displays the studied compositions microstructures confirming the good distribution of Ag NPs (average size 9.2 nm) upon Nd_2_O_3._ The results interpretations boost usage of such composites in sensors industries, and water treatment. The research team highlights the significance of investigating the prolonged stability and resilience of the Ag/Nd_2_O_3_ nano-composite under actual environmental conditions. Assessing the material’s performance under various environmental conditions, such as temperature, pH, and humidity, would provide useful insights for its practical utilization. Moreover, the authors propose investigating the potential of the Ag/Nd_2_O_3_ nano-composite in other domains apart from antibacterial applications. For example, exploring its photocatalytic qualities for water treatment or its sensing capabilities for environmental monitoring might provide new opportunities for its use in these domains.

## Data Availability

The datasets used and/or analysed during the current study available from the corresponding author on reasonable request.
